# *QuickStats*: Age-Adjusted Percentage* of Adults Aged ≥45 Years Who Were Limited in Any Way Because of Difficulty Remembering or Periods of Confusion,^†^ by Race/Ethnicity^§^ — United States, 2014–2016^¶^

**DOI:** 10.15585/mmwr.mm6643a10

**Published:** 2017-11-03

**Figure:**
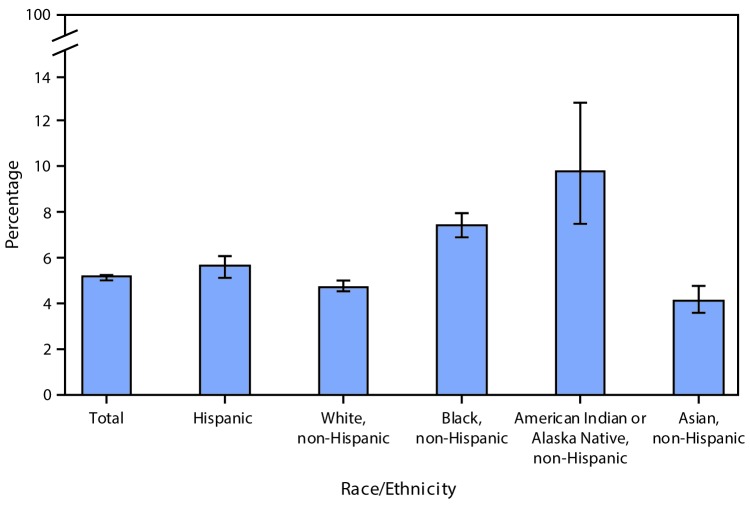
Overall, 5.1% of adults aged ≥45 years were limited in any way because of difficulty remembering or periods of confusion. The percentage of adults experiencing this limitation was highest among non-Hispanic American Indian/Alaska Native adults (9.8%) and non-Hispanic black adults (7.4%), followed by Hispanic adults (5.6%), non-Hispanic white adults (4.7%), and non-Hispanic Asian adults (4.1%).

**Source:** National Health Interview Survey, 2014–2016 data. https://www.cdc.gov/nchs/nhis.htm.

